# Exploration of double-dart injection technique as a supplemental application for remote drug delivery system for zoo and wild animals

**DOI:** 10.14202/vetworld.2022.622-626

**Published:** 2022-03-19

**Authors:** Rattapan Pattanarangsan, Pawinee Kulnanan, Watcharapong Mitsuwan, Tuempong Wongtawan

**Affiliations:** 1 Akkharatchakumari Veterinary College, Walailak University, Nakhon Si Thammarat, 80160, Thailand; 2 One Health Research Center, Walailak University, Nakhon Si Thammarat, 80160, Thailand; 3 Center of Excellence in Innovation of Essential Oil, Walailak University, Nakhon Si Thammarat, 80160, Thailand

**Keywords:** double-dart technique, free-ranging animals, remote drug delivery, wildlife

## Abstract

**Background and Aim:**

Remote drug delivery has become an essential tool for safely delivering medication and vaccines to free-ranging, non-domestic, or dangerous animals. All dart guns currently use a single dart per injection, and it might occasionally be not practical with large animals. Shooting the dart more than once on an animal may cause flight, injury, stress, and ultimately unsuccessful delivery. Furthermore, purchasing many dart guns and hiring and training more staff may be unfeasible in developing countries. Therefore, employing the double-dart injection technique may help reduce the cost of operation, save time for capturing animals, minimize stress and injury, and improve animal welfare. The objectives of this study were to test the possibility of using the double-dart injection technique and optimizing the guidelines for this procedure.

**Materials and Methods:**

A standard brand-calibrated darting rifle was used to deliver the darts to the target board constructed from paper, polypropylene, and ethylene-vinyl acetate foam. The shot stage and shooter were fixed, and the shooting range was 5-20 m. The pressure of the gun was varied according to a company’s recommendation. The single dart (control dart) was first shot to the target point, and then the double darts were shot 3 times for each condition. The experiment was done in the field with no wind. The inclusion criteria were that two darts must hit the target and not penetrate the target board deeply. The distances between the control dart and double darts (first and second darts) and between each dart of the double darts were measured, and the standard curve graphs and formulas were created.

**Results:**

The results showed that the distance between the control dart and the double darts was shortened as the pressure was increased. All double-dart injections hit the target below the control dart. We were able to create many formulas to predict the optimal gun pressure and aim point for double-dart injection in each shot range. It usually requires more pressure settings than a single-dart injection, particularly the long shot range. It also needs to aim the target point above the original point.

**Conclusion:**

Double-dart injection technique can be used efficiently in 5-20 m distance, and it usually requires increasing the pressure from the company’s recommendation and adjusting the injecting point.

## Introduction

Remote drug delivery has become an important tool for allowing the capture and safe delivery of medication and vaccines to free-ranging animals, zoo animals, marine animals, and wildlife. This method ensures safety for veterinarians, zookeepers, game captures, and wildlife researchers. [1,2]. The two main types of remote injection equipment commonly used in zoos are blowpipes and dart guns. Blowpipes have been used by local people for more than a century to capture animals and fight [3]. It has been applied to use with an auto-syrinx dart to deliver the drug to many animals with a shorter shooting range than the dart gun [4]. Dart gun injection is a widely accepted and routine procedure for administering anesthesia to various animals, including dangerous animals [5,6]. In large animals such as elephants, the dart gun injection is vital to manage the violence caused by musth elephant and wild elephant-human conflicts. Wild elephant-human conflicts are found in many countries, including Thailand, leading to human and elephant injury and death, as well as property damage [7]. Injections of tranquilizers and anesthesia have been used to calm down and move these animals [8,9].

When working with large or mega animals, the volume of the drug usually increases accordingly with the size of the animal. There are circumstances in which there is a lack of availability of large-volume darts for various reasons in the field. The immediate solution is to put the drug into two darts and shoot twice. However, the first shot can lead the animal to become frightened and escape, creating stress and leading to injury or death in the worst-case scenario [10]. This problem may be fixed by having two guns and two professional staff, but this strategy would cost more money to buy guns, hire more staff, and do training. Hence, this solution is usually impractical in developing countries [11]. The double-dart injection technique might be an additional or alternative technique helping field officers reduce loss and injury in specific conditions that may happen. Occasionally, a lack of large-volume darts may arise, creating a life-or-death emergency for animals and humans. Furthermore, this technique could assist with treating sick animals that require two kinds of drugs simultaneously. Therefore, knowing the double-dart injection technique with proper guidelines can help manage this situation.

We hypothesized that double-dart injection might be possible, but the experiment and protocol have not been established yet. The objectives of this study were to test the possibility of using double-dart gun injection and then to optimize the guidelines for this procedure.

## Materials and Methods

### Ethical approval

This article does not contain any studies with human participants or animals performed by any of the authors. So, it does not require ethical approval.

### Dart gun

In this experiment, the JM standard Dan-Inject rifle (Dan-Inject ApS, Kolding, Denmark), one of the popular injection guns, was used to deliver the dart. According to the manufacture’s information, the length of the gun is 105 cm, weighing 2.9 kg, with an 11 mm bore barrel, using CO_2_ as the gas pressure. The telescopic sight is 1.5-4.5×32 mm, and the effective range is 1-40 m. The target is pointed with a crossed hair type telescope. The maximum pressure of this dart gun is 16 bar. In this study, the maximum gun pressure used was 11 bar, and the dart size was 3 mm.

### Target

**Figure-1:**
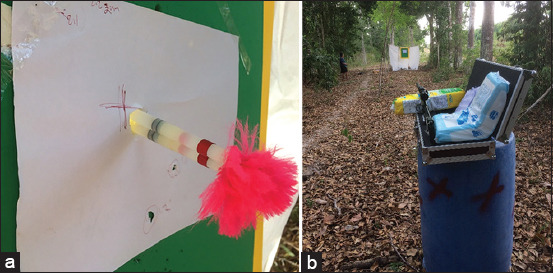
Target and shooting stage setting. (a) An A4 paper was marked with a cross for targeting. The green material was ethylene-vinyl acetate foam, and the yellow board was polypropylene. (b) An injection gun was stationed on stage for each shot.

The target was constructed from A4 paper with a cross mark attached to a polypropylene board and ethylene-vinyl acetate foam. The shooting stage was fixed to minimize human effects. The target distance (shot range) was 5-20 m (Figure-1).

### Experimental design

The injection was done by one person to reduce the variability between people. A gun was stationed on stage for each shot. The pressure of the gun was varied based on the company’s recommendation (for single-dart injection) to identify the optimal pressure for each distance (Table-1). At each distance, the single dart was first shot to the target point to validate the gun (control); then, the double darts were shot 3 times for each experimental condition. The experiment was done in the field with no wind, and wind flags were used to detect wind.

**Table-1:** Distance of target (shot range) and the gun pressure

Distance (m)	Pressure setting (bar)						
5	1	2	3*	4	5	6	7
10	2	3	4*	5	6	7	8
15	3	4	5*	6	7	8	9
20	5	6	7*	8	9	10	11

* Company’s recommendation for single dart injection

### Criteria

The inclusion criteria were that two darts must hit the target and not penetrate the target board deeply. For exclusion criteria: (1) A scenario of “too-low impact,” one or two darts did not hit the target; (2) a scenario of “too-high impact,” the dart deeply penetrated the target board; and (3) the two darts pierced away from each other by more than 15 cm which could be a miss shot in shooting large animals up to deer size.

### Measurement

The distance between the control dart (single-dart injection) and double darts (first and second darts), and the distance between each dart of double-dart injection were measured. The distance between the first dart and the control dart was called D1-C. The distance between the second dart and the control dart was called D2-C. The distance between the first dart and the second dart was called D1-2. Standard curve graphs and formulas were created using Microsoft Excel (Microsoft Corporation, Washington, USA).

## Results and Discussion

The conditions that meet our criteria are shown in Table-2. The optimal pressure for the 5 m shot range was 2-5 bar; for the 10 m shot range, it was 4-8 bar; for the 15 m shot range, it was 6-9 bar; and for the 20 m shot range, it was 9-11 bar. The company’s recommendation for single-dart injection can be used for double injections at a shooting range of 5-10 m. For a shot range more than 15 m, the pressure must be adjusted above the manufacturer’s suggestion. Some rifle guns can have a shot range up to 100 m, but it is recommended to shot less than 10 m, if possible, particularly small and medium size animals to minimize the tissue damage [4,12].

**Table-2:** The conditions that pass the criteria for double dart injection

Distance (m)	Pressure setting (bar)						
5	1^X^	2*	3*	4*	5*	6^Y^	7^Y^
10	2^X^	3^X^	4*	5*	6*	7*	8*
15	3^X^	4^X^	5^X^	6*	7*	8*	9*
20	5^X^	6^X^	7^X^	8^X^	9^Z^	10^Z^	11^Z^

* Successful criteria

X Exclusion criteria: too light, not piercing

Y Exclusion criteria: too high, impact force

Z Exclusion criteria: the distance between each dart was more than 15 cm

**Figure-2:**
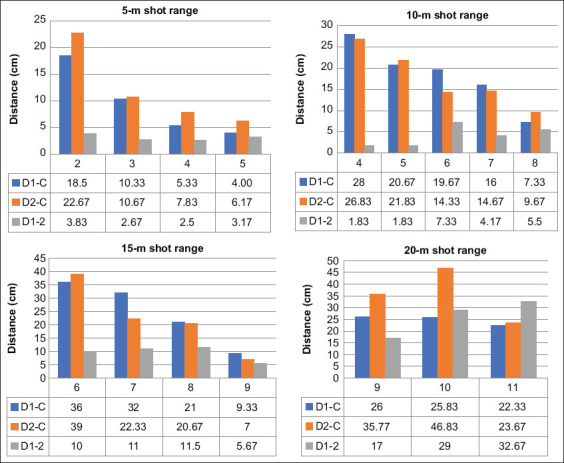
The distance between each dart for different shot ranges (5-20 m). The first dart and the control dart (D1-C), the second dart and the control dart (D2-C), and the first dart and the second dart (D1-2).

The distances between the first dart and the control dart (D1-C), between the second dart and the control dart (D2-C), the first dart, and between the second darts (D1-2) in each shot range are shown in Figure-2. The distance between the control dart and the double darts was shortened as the pressure was increased. The D1-2 value for the 20 m shot range was higher than the D1-2 value for other shorter shot ranges (5-12 m); the D1-2 value for the 20 m shot range was between 17 and 32 cm, whereas the D1-2 value for other shot ranges was <12 cm. Notably, all double-dart injections hit the target below the control darts. Shooting drugs from a distance greater than 20 m protects the shooter from huge and dangerous animals such as elephants, bears, and rhinoceros while maintaining an acceptable 32-cm gap between two darts [5,8].

**Figure-3:**
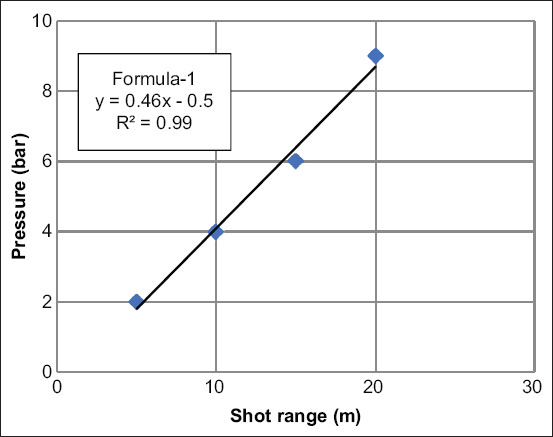
Standard curve and formula of recommended minimum pressure for double-dart injection (Y=pressure setting and X=the shooting range) in each shot range.

From Table-2, the minimal pressures that can be used for double dart injection at each shot range were plotted as a standard curve graph (Figure-3). Then, the formula was established to predict the recommended minimum pressure for injecting in desired shot range (formula-1). The Formula-1 was Y (pressure setting)=0.46X (the shooting range)-0.5. The coefficient of determination (R square; R^2^) was 0.99. We recommend using the least amount of pressure feasible to minimize the harm caused by kinetic energy and projectiles from darts and gunshots [11,13].

**Figure-4:**
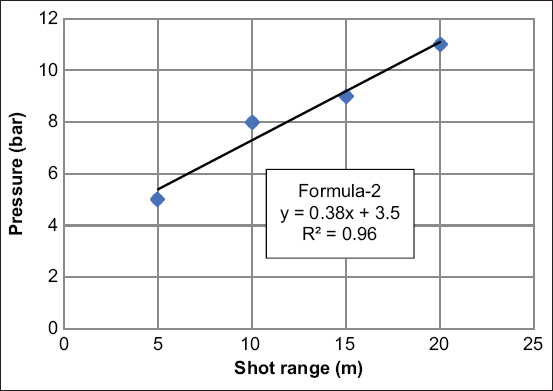
Standard curve and formula of optimal pressure for double-dart injection (Y=pressure setting and X=the shooting range) in each shot range.

We also plotted the graph to represent the optimal pressure and distance of the shot range (Figure-4). The optimal pressure was selected by the pressure that created the smallest distance between the control dart and the double darts (the average of DC-1 and DC-2) and was calculated into the Formula-2 to predict the optimal pressure for each desired shot range (formula-2). This Formula-2 was Y (gun pressure)=0.38X (the shot range)+3.5. The coefficient of determination (R square; R2) was 0.96.

**Figure-5:**
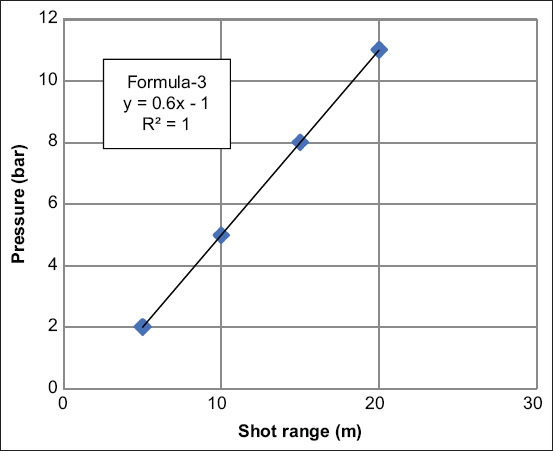
Standard curve and formula of optimal pressure for double-dart injection in each shot range (Y=gun pressure and X=the shot range). The target point (double darts) was 20 cm lower than the original point (control dart).

**Figure-6:**
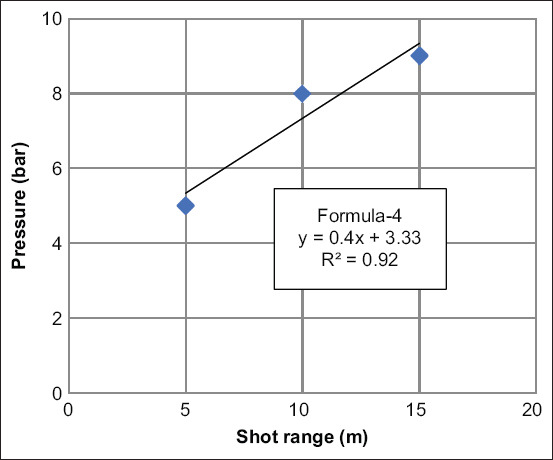
Standard curve and formula of optimal pressure for double-dart injection, the target point was about 7 cm lower than control dart (Y=gun pressure and X=the shot range). The target point (double darts) was 9 cm lower than the original point (control dart).

Because of the fact that the double-dart injection was always hit the target below the control dart injection (original target point) and knowing the predicted distance between the control dart and the double darts could assist the shooter in predicting the injection location of double darts that can be used to adjust the injecting aim point. This strategy will be safer for the animals and more effective for the shot. Therefore, we chose two fixed distances (20 cm and <9 cm) between the control dart and the double darts, a gun pressure capable of producing a similar distance to create graphs and formulas that could predict the distance between the control and the double darts (Figures-5 and 6). For the 20 cm distance between the control dart and the double darts, the shot ranges were 5, 10, 15, and 20 m. The gun pressures were 2, 5, 8, and 11 bar, respectively. Thus, the formula for predicting the distance of double dart from the original target point (formula-3) was Y (pressure setting)=0.6X (the shooting range)-1, the coefficient of determination (R square; R2) was 1, which is perfect for the linear model (Figure-5).

For <9 cm distance between the control dart and the double darts, the shot ranges were 5, 10, and 15 m, and the gun pressures were 4, 8, and 9 bar, respectively. Hence, the formula for predicting the distance of double dart from the original target point (formula-4) was Y (gun pressure)=0.4X (shot range)+3.33, the coefficient of determination (R square; R2) was 0.92 (Figure-6). While a small distance between two darts is preferable to ensure that both darts strike the target, it demands high pressure, resulting in additional injuries to the animals [11,13].

All formulas we created had the coefficient of determination (R square; R2) higher than 0.9, which was quite strong, suggesting that the predicting model was highly precise.

## Conclusion

We propose that the double-dart injection technique can be utilized to deliver medication remotely. The setup entails increasing the gun pressure over the manufacturer’s recommendation and adjusting the aiming point upward from the original target. The optimal pressure and the adjusting aim point for each short range can be predicted using the formula we created. Although this study proves that double dart injection is feasible, it still needs to be validated in the field and with different gun types.

## Authors’ Contributions

RP: Designed and performed the study, analyzed the data, and received the research grant. TW: Designed and consulted the study, contributed to the analysis and interpretation of data, wrote the first draft manuscript, and received the research grant. PK: Performed the study and analyzed the data. WM: Analyzed the data and wrote the first draft of the manuscript. All authors have read and approved the final manuscript.
